# Multicenter Surveillance of Antimicrobial Resistance among Gram-Negative Bacteria Isolated from Bloodstream Infections in Ghana

**DOI:** 10.3390/antibiotics12020255

**Published:** 2023-01-27

**Authors:** Eric S. Donkor, Khitam Muhsen, Sherry A. M. Johnson, Fleischer C. N. Kotey, Nicholas T. K. D. Dayie, Patience B. Tetteh-Quarcoo, Edem M. A. Tette, Mary-Magdalene Osei, Beverly Egyir, Nicholas I. Nii-Trebi, Godfred Owusu-Okyere, Alex Owusu-Ofori, Yonatan Amir, Saritte Perlman, Perdita Hilary Lopes, Adjo Mfodwo, Nicola C. Gordon, Louise Gresham, Mark Smolinski, Dani Cohen

**Affiliations:** 1 Department of Medical Microbiology, University of Ghana Medical School, Accra 00233, Ghana; 2 Department of Epidemiology and Preventive Medicine, School of Public Health, Sackler Faculty of Medicine, Tel Aviv University, Tel Aviv 6139001, Israel; 3 Middle East Consortium on Infectious Disease Surveillance (MECIDS), Jerusalem 9149302, Israel; 4 School of Veterinary Medicine, University of Ghana, Accra 00233, Ghana; 5 Department of Community Health, University of Ghana Medical School, Accra 00233, Ghana; 6 Department of Bacteriology, Noguchi Memorial Institute for Medical Research, University of Ghana, Accra 00233, Ghana; 7 Department of Medical Laboratory Sciences, School of Biomedical & Allied Health Sciences, College of Health Sciences, University of Ghana, Accra 00233, Ghana; 8 National Public Health Reference Laboratory, Accra 00233, Ghana; 9 Department of Clinical Microbiology, Kwame Nkrumah University of Science and Technology, Kumasi 00233, Ghana; 10 Directorate of Laboratory Services, Komfo Anokye Teaching Hospital, Kumasi 10900, Ghana; 11 Mott MacDonald, Accra 00233, Ghana; 12 Mott MacDonald, London N11 3JB, UK; 13 Ending Pandemics, San Francisco, CA 94102, USA

**Keywords:** Gram-negative bacteria, non-typhoidal *Salmonella*, *Klebsiella pneumoniae*, bloodstream infections, antibiotic resistance, multidrug resistance

## Abstract

Background: Antimicrobial resistance (AMR) in Gram-negative bacteria-causing bloodstream infections (BSIs), such as *Klebsiella pneumoniae* and non-typhoidal *Salmonella* (NTS), is a major public health concern. Nonetheless, AMR surveillance remains scarce in sub-Saharan Africa, where BSI treatment is largely empirical. The aim of the study was to determine the distribution and AMR patterns of BSI-causing NTS, *K. pneumoniae*, and other Gram-negative bacteria in Ghana. Methods: A cross-sectional study was conducted between April and December 2021 at eleven sentinel health facilities across Ghana as part of a pilot study on the feasibility and implementation of the human sector AMR surveillance harmonized protocol in sub-Saharan Africa. Gram-negative bacteria recovered from blood specimens of febrile patients were identified using MALDI-TOF and evaluated for antimicrobial resistance using the BD Phoenix M50 analyzer and Kirby-Bauer disc diffusion. The Department of Medical Microbiology at the University of Ghana served as the reference laboratory. Results: Out of 334 Gram-negative blood isolates, there were 18 (5.4%) NTS, 85 (25.5%) *K. pneumoniae*, 88 (26.4%) *Escherichia coli*, 40 (12.0%) *Acinetobacter baumannii*, 25 (7.5%) *Pseudomonas aeruginosa*, and 77 (23.1%) other Gram-negative bacteria. As a composite, the isolates displayed high resistance to the antibiotics tested—amoxicillin (89.3%), tetracycline (76.1%), trimethoprim-sulfamethoxazole (71.5%), and chloramphenicol (59.7%). Resistance to third-generation cephalosporins [ceftriaxone (73.7%), cefotaxime (77.8%), and ceftazidime (56.3%)] and fluoroquinolones [ciprofloxacin (55.3%)] was also high; 88% of the isolates were multidrug resistant, and the rate of extended-spectrum beta-lactamase (ESBL) production was 44.6%. Antibiotic resistance in *K. pneumoniae* followed the pattern of all Gram-negative isolates. Antibiotic resistance was lower in NTS blood isolates, ranging between 16.7–38.9% resistance to the tested antibiotics. Resistance rates of 38.9%, 22.2%, and 27.8% were found for cefotaxime, ceftriaxone, and ceftazidime, respectively, and 27.8% and 23.8% for ciprofloxacin and azithromycin, respectively, which are used in the treatment of invasive NTS. The prevalence of multidrug resistance in NTS isolates was 38.9%. Conclusions: Multicenter AMR surveillance of Gram-negative blood isolates from febrile patients was well-received in Ghana, and the implementation of a harmonized protocol was feasible. High resistance and multidrug resistance to first- or second-choice antibiotics, including penicillins, third-generation cephalosporins, and fluoroquinolones, were found, implying that these antibiotics might have limited effectiveness in BSI treatment in the country. Continuation of AMR surveillance in Gram-negative blood isolates is essential for a better understanding of the extent of AMR in these pathogens and to guide clinical practice and policymaking.

## 1. Introduction

Bloodstream infections (BSIs) remain a major public health threat globally, despite significant management and therapeutic advancements [1,2,3,4,5,6,7,8,9,10,11,12]. A meta-analysis showed regional differences in the prevalence of community-onset BSIs, with a median prevalence of 7.3% in the Americas, 2.9% in Europe, 7.3% in Asia, and 14.6% in Africa [12]. BSIs are mostly caused by bacteria, with potential temporal and regional variation in the relative proportion of the pathogens causing BSI [12,13,14,15]. Gram-negative bacteria such as *Escherichia coli* (*E. coli*), non-typhoidal *Salmonella* (NTS), and *Klebsiella pneumoniae* (*K. pneumoniae*) are among the common etiological agents [12,14,16,17,18]. *E. coli* and *K*. *pneumoniae* are also leading causes of healthcare-associated BSIs [19,20]. NTS is a leading cause of BSIs, mainly in sub-Saharan Africa [17,21,22,23], where HIV infection and malnutrition are common [21,23,24]. Although *K*. *pneumoniae* BSIs are traditionally opportunistic in immunocompromised people, they are also being increasingly encountered among the immunocompetent due to the emergence of hypervirulent and resistant strains [25,26,27]. Evidence regarding the etiological role of *K. pneumoniae* in sepsis/bacteremia in sub-Saharan Africa is limited, and the treatment of BSI remains largely empirical. Both NTS and *K. pneumoniae* are listed among the Global Antimicrobial Surveillance System (GLASS) priority pathogens [28].

Importantly, treatment of Gram-negative bacteria-caused BSIs (and other bacterial infections) has become more challenging due to antimicrobial drug resistance (AMR), a major public health menace in sub-Saharan Africa [29]. In 2019 alone, 1.27 million deaths were attributed to bacterial AMR globally, with western sub-Saharan Africa having the highest death rates attributable to AMR: 27.3 per 100,000 [30]. It was estimated that by 2050, the average annual number of deaths attributed to AMR will reach 10 million if nothing is done about the AMR problem [31,32].

Surveillance of AMR is essential for monitoring the extent and spread of AMR and the effectiveness of interventions aimed at mitigating its burden [33]. Surveillance of AMR underpins the availability of high-quality data to guide policymaking toward AMR control and prevention nationally, regionally, and globally [31,34]. Therefore, harmonized epidemiological and microbiological surveillance protocols are crucial across these levels. Quantifying the burden of AMR is challenging due to the multidimensional nature of the issue [31,34,35]. Furthermore, these challenges are amplified in low-and middle-income countries given the limited resources, infrastructure, trained manpower, lack of point-of-care diagnostics, and sometimes limited commitment from policymakers [31,34]. Addressing AMR while following the One Health approach is highly desirable [34]. Such efforts are particularly needed in sub-Saharan Africa, a highly affected region by AMR and its drivers.

Accordingly, we developed an AMR surveillance protocol in the framework of the Fleming Fund regional program and as part of a pilot project themed “One Health Surveillance of AMR in Non-Typhoidal *Salmonella*, *Klebsiella pneumoniae*, and Other Gram-Negative Bacteria”. Herein, we describe the implementation of the human sector AMR surveillance protocol in a multicenter study across Ghana and provide preliminary results on AMR patterns in Gram-negative bacteria (especially NTS and *K. pneumoniae*) from patients with BSIs. Of note, like many countries in sub-Saharan Africa, nationwide AMR surveillance in Ghana is limited, although efforts to implement such programs are in place, including the development of a National Action Plan (NAP) and investment in training and infrastructure. A prior nationwide AMR surveillance program implemented in Ghana over a six-month period involving 1598 samples and 24 laboratories mostly analyzed outpatient specimens (60.3%) and urine (38.6%) samples, while only 100 (6.3%) blood cultures were included [36]; thus evidence on AMR among patients with severe invasive disease remains limited. To address this gap, we determined the distribution and AMR patterns of BSI-causing NTS, *K. pneumoniae*, and other Gram-negative bacteria in Ghana.

## 2. Results

### 2.1. Characteristics of the Study Samples

A total of 334 blood cultures tested positive for Gram-negative bacteria. The isolates were distributed across all sentinel sites, with 27.8% from Korle Bu Teaching Hospital, 16.8% from Greater Accra Regional Hospital, and 12.3% from Eastern Regional Hospital. The other sentinel sites contributed fewer isolates (Table 1). The majority of the isolates (66.2%) were obtained from the Greater Accra Region. Data on age and gender were missing for 53.3% and 41.7% of the patients, respectively. Among those with complete information on gender, 49.4% were females. The age of the patients ranged between 0–86 years, with a median of 6 years. Children aged less than five years comprised 47.2% of the patients with complete information on age (*n* = 180), while 12.2%, 30.6%, and 10.0% of the patients were 5–19, 20–59, and 60+ years, respectively. Most patients (98.5%) had sepsis/bacteremia, two patients (0.6%) had urosepsis, and three (0.9%) had other conditions.

### 2.2. Comparison between the Bacteria Isolated at the Sentinel and Reference Laboratories

The reference laboratory identified more isolates as *Acinetobacter baumannii* and *K. pneumoniae* than the sentinel laboratories (~difference +5%). The difference for other pathogens was smaller. The agreement between the reference and sentinel laboratories was significant. Cohen’s Kappa coefficient was 0.669 for the agreement between the reference and sentinel laboratories regarding the isolation of *A. baumannii*, but it was higher for other pathogens (ranging from 0.796 for *K. pneumoniae* to 0.864 for *Salmonella* spp.) (Table 2).

**Figure 1:**
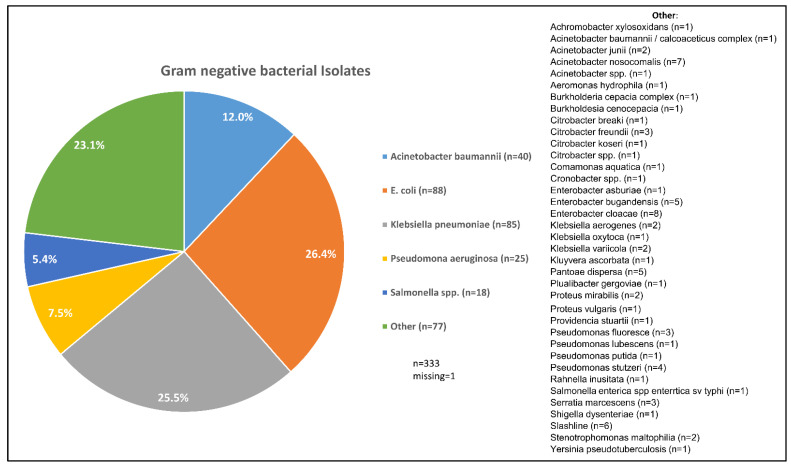
Distribution of the Gram-negative blood isolates as detected at the reference laboratory.

Based on the detection in the reference laboratory, there were 18 (5.4%) *Salmonella* spp. blood isolates, 85 (25.5%) *K. pneumoniae*, 88 (26.4%) *E. coli*, 40 (12.0%) *A. baumannii*, 25 (7.5%) *P. aeruginosa*, and 77 (23.1%) other Gram-negative bacteria (Figure 1).

### 2.3. Antimicrobial Drug Susceptibility of the Gram-Negative Blood Isolates

Considering all Gram-negative blood isolates, antibiotic resistance exceeded 50.0% for 10 out of the 13 tested antibiotics; the antimicrobials against which lower than 50% resistance rates were recorded were meropenem, azithromycin, and ertapenem. Multidrug resistance was found in 88.0% and ESBL production in 44.6% of the isolates. Antibiotic resistance in *K. pneumoniae* isolates was similar to that of all Gram-negative isolates; *Salmonella* spp. exhibited a lower prevalence of antibiotic resistance. *A. baumannii*, *E. coli*, and *P. aeruginosa* generally had a similar AMR pattern to that of *K. pneumoniae,* with a few exceptions; the prevalence of resistance to gentamicin and ciprofloxacin was higher in *K. pneumoniae*. In addition, resistance to carbapenems was much higher in *P. aeruginosa* isolates than in *K. pneumoniae*, NTS, and *E. coli* isolates. *A. baumannii* had similar high resistance rates to ertapenem (90.0%), but meropenem resistance was comparable to the resistance in the other isolates. The resistance rate to nalidixic acid was higher in *E. coli* (77.3%) and *P. aeruginosa* (92.0%) than it was in the other isolates (16.7–58.3%) (Table 3). A detailed distribution of the prevalence of AMR profile (resistant, intermediate, or susceptible) of these bacterial pathogens appears in Supplementary Tables S1–S3.

There was no significant difference in the prevalence of MDR bacteria according to sentinel site, city, region, gender, or age (Table 4).

## 3. Discussion

In this nationwide pilot study, we determined the distribution and AMR patterns of BSI-causing Gram-negative bacteria across selected health facilities in Ghana. The main finding of this study is the feasibility and success of the pilot study of national AMR surveillance in Ghana.

Generally, nationwide AMR surveillance is especially challenging in low- and middle-income countries given the required awareness, knowledge, multi-sectoral collaboration, and required infrastructure and expertise on the one hand and the limited resources and frail infrastructure on the other hand. Herein, we demonstrated that in Ghana, as a model of low-middle-income countries, such a surveillance program is, in fact, practicable and provisional to the availability of adequate support, infrastructure, expertise, and commitment.

Our pilot was well received by the Ghanaian collaborative sentinel sites that provided 334 Gram-negative isolates from both sexes and all age groups during an intensive nine months of sample collection. There was good agreement in the detection of the Gram-negative blood isolates between the sentinel microbiology laboratories and the reference laboratory, although some minor discrepancies existed. This highlights the important role of the reference laboratory in supporting and coordinating nationwide AMR surveillance programs.

The leading Gram-negative pathogens that were isolated from blood in this study were *E. coli* (26.4%), followed by *K. pneumoniae* (25.5%), *A. baumannii* (12.0%), and *P. aeruginosa* (7.5%). NTS accounted for 5.4% of the isolates, while other pathogens were detected in 23.1%. While *E. coli*, *P. aeruginosa* and NTS were shown to be leading etiological agents of BSI caused by Gram-negative bacteria [13,36,37], the emergence of *K. pneumoniae* in our study as a leading cause of BSI is alarming. Other studies from sub-Saharan Africa have also documented the emergence of *Klebsiella* species, mainly *K. pneumoniae,* in Gram-negative BSIs [13,38,39]. The traditional human gut-colonizing *K. pneumoniae* is emerging as a hyper-virulent pathogen globally [26,27]. Our study adds new knowledge on its etiological role in sepsis/bacteremia in sub-Saharan Africa.

Considering all Gram-negative blood isolates, we found high resistance to antibiotics included in the WHO Essential Medicines List in the “access group”, that is, first- or second-choice antibiotics that offer the best therapeutic value while minimizing the potential for resistance [40]. This included 89.3% resistance to amoxicillin, 76.1% to tetracycline, 71.5% to trimethoprim-sulfamethoxazole, and 59.7% to chloramphenicol. Ampicillin, trimethoprim-sulfamethoxazole, and chloramphenicol are recommended as empiric first-choice treatments for many infectious syndromes, including invasive infections. A prior nationwide study from Ghana that spanned over a six-month period included 1598 clinical bacterial isolates, but only 100 of these were from blood [36]. Another study from Ghana included 49 Gram-negative MDR blood samples isolated from BSI patients [37]. Both studies showed similar high antibiotic resistance rates against ampicillin, trimethoprim-sulfamethoxazole, and chloramphenicol, in Gram-negative pathogens [36,37]. Similar findings were reported from Malawi [13]. Collectively, these results indicate the limited effectiveness of these antibiotics.

Another finding of concern in our study is the high prevalence rates of resistance to third-generation cephalosporins; ceftriaxone (73.7%), cefotaxime (77.8%), and ceftazidime (56.3%). A recent systematic review of studies published between 1990 and 2019 assessed the prevalence of third-generation cephalosporins susceptibility of *E. coli*, *Klebsiella,* and *Salmonella* BSI in sub-Saharan Africa showed a median resistance of 18.4%, 54.4%, and 1.9%, respectively [41]. Other studies from Ghana reported high resistance rates to third-generation cephalosporins in Gram-negative pathogens, in agreement with our results [36,37]. A study from Malawi reported high rates (>90%) of resistance to third-generation cephalosporins (Ceftriaxone) for *Klebsiella* and *Acinetobacter* spp. isolated from blood during 2013–2017 but much lower rates for *E. coli* (28%) and NTS (1%) [13].

We also found a high prevalence of ESBL producers (44.6%) among Gram-negative blood isolates of BSI patients, similar to that reported in another study conducted in Ghana—49.3% [36]. A multi-country study that included Gram-negative blood isolates from patients with BSI in sub-Saharan Africa showed that among 423 *Enterobacteriaceae* isolates, phenotypically, 12.1% isolates exhibited ESBL activity, and genotypically, 9.3% yielded a PCR amplicon for one or more of the screened ESBL genes. This study also showed that ESBL prevalence varied across bacterial pathogens: 45.5% for *Klebsiella* spp., 5.7% for *E. coli*, 16.2% for *Acinetobacter* spp., and 1.3% for NTS [39].

Resistance to ciprofloxacin of the Fluoroquinolone family was also high (55.3%). These antibiotics are in the “watch group” of the WHO categories, i.e., first- or second-choice antibiotics, only indicated for a specific, limited number of infective syndromes, more prone to be a target of antibiotic resistance, and thus prioritized as targets of stewardship programs and monitoring [40]. Remarkably, 88% of the blood isolates were multidrug resistant.

Antibiotic resistance in *K. pneumoniae* blood isolates followed the pattern of all Gram-negative isolates. Collectively, these results are especially alarming and require further verification in surveillance of a longer duration to robustly estimate the resistance rates to these essential antibiotics. Interestingly, antibiotic resistance was lower in the NTS blood isolates, ranging between 16.7% and 38.9% resistance to the tested antibiotics. Nonetheless, resistance rates of 38.9%, 22.2%, and 27.8% were found for cefotaxime, ceftriaxone, and ceftazidime, respectively, 27.8%, and 23.8% for ciprofloxacin and azithromycin, respectively, which are used in the treatment of invasive NTS. The prevalence of multidrug resistance in NTS isolates was 38.9%. Tack et al. [42], in a meta-analysis that assessed AMR in invasive NTS in sub-Saharan Africa, defined multidrug resistance based on the antibiotic, ampicillin, trimethoprim-sulfamethoxazole, or chloramphenicol. They estimated a prevalence of multidrug resistance of 75% in invasive NTS since 2001 across all regions in sub-Saharan Africa. The pooled estimate of resistant NTS to third-generation cephalosporins reached up to 5% after 2010 [42], while in our study, this figure was substantially higher (20–38%). Tack et al. [42] reported 1% resistance to fluoroquinolone and limited evidence of resistance to azithromycin, while in our study, this estimate exceeded 20%.

Carbapenems, broad-spectrum antibiotics, are classified by the WHO as critically important antimicrobials for human medicine [43]. Mechanisms of resistance to carbapenems involve the production of β-lactamases, efflux pumps, and mutations that change the expression and or function of porins and penicillin-binding proteins [44]. Resistance to these drugs was relatively lower compared with other tested antibiotics (28.0% for meropenem and 44.4% for ertapenem in all Gram-negative isolates. However, these rates were much higher in *P. aeruginosa* (52.0–84%) and *A. baumannii* isolates (30.8–90.0%). A recent study from Ethiopia reported 58.8% resistance to meropenem in *A. baumannii* blood isolates, 17.1% in *K. pneumoniae,* and only 5.9% in *P. aeruginosa* and 1.7% in *E. coli* isolates [45]. A study of neonates hospitalized in neonatal intensive care units in Ghana showed 4.4% carbapenem resistance in *K. pneumoniae*-positive blood cultures [46]. The emergence of carbapenem resistance was described in other regions in sub-Saharan Africa [47]. Carbapenems resistance is a main clinical and public health concern, given the increased mortality risk associated with infections resistant to these drugs [48] and the frail medical system in limited-resources settings.

The main limitations of our study include the short duration of samples and data collection, which might have missed seasonal or year-to-year variation in the occurrence of specific BSIs, limited clinical information, and missing information on the demographics of a high proportion of the study subjects. Nonetheless, we successfully achieved the primary goal of this pilot study of assessing the feasibility and practicality of nationwide AMR surveillance of BSIs in Ghana. The main strengths of the study include nationwide surveillance using harmonized clinical and microbiological protocols across all sentinel laboratories and central coordination and quality control that was undertaken by a local reference microbiology laboratory.

## 4. Materials and Methods

### 4.1. Study Design, Sites, and Sampling

This study was part of the Fleming Fund regional project on AMR surveillance in sub-Saharan Africa, during which a mapping and gap analysis phase was undertaken. In the framework of the project, we developed a generic AMR surveillance protocol for NTS and *K. pneumoniae* following the One Health approach. The pilot implementation of the protocol was conducted in Ghana. The study had an international steering committee that provided oversight of the project.

A cross-sectional study was conducted between April and December 2021 at selected geographically diverse health facilities across Ghana. The Department of Medical Microbiology, University of Ghana Medical School (UGMS), served as the reference laboratory for this surveillance, along with several sentinel sites—37 Military Hospital, Greater Accra Regional Hospital, Eastern Regional Hospital, Effia-Nkwanta Regional Hospital, Komfo Anokye Teaching Hospital, Korle Bu Teaching Hospital, Princess Marie Louise Children’s Hospital, St. Patrick’s Hospital, Volta Regional Hospital, and two private laboratories (Table 5). The selection of the sentinel sites was determined based on the availability of suitable facilities for bacteriological culture, high patient turnout, and adequate geographical coverage of the country. The study was coordinated at the Department of Medical Microbiology, UGMS, which functions as the national AMR reference laboratory, together with three sentinel sites—Korle Bu Teaching Hospital, Eastern Regional Hospital, and Volta Regional Hospital—were supported and upgraded by the Ghana Fleming Fund country grant, thus ensuring adequate laboratory infrastructure.

Gram-negative bacterial isolates recovered from blood specimens of patients presenting to the sentinel sites with febrile illness during the study period were collated and analyzed. Febrile illness was defined as having a body temperature of ≥38.0 °C that began three days before the visit to the sentinel site, according to the patient/guardian’s report or review of the medical chart. Basic demographic information was recorded.

### 4.2. Laboratory Analysis

Training for harmonization of the laboratory methods across the study sites was undertaken by the team of the Medical Microbiology Department at the University of Ghana.

Blood cultures were performed at sentinel site laboratories using a commercially available automatic blood culture incubation system (BD), according to the manufacturer’s instructions. Where bacterial growth was detected, Gram staining was performed, and subcultures were done on 5% sheep blood agar, chocolate agar, and MacConkey agar plates (Oxoid, Basingstoke, Hants, UK) and incubated for 18–24 h at 35 ± 2 °C in ambient air (for the MacConkey plates) and for 18–24 h at 35 ± 2 °C in 5% CO_2_ (for blood and chocolate agar plates). Bacterial isolates were identified using routine biochemical methods (such as lactose, oxidase, citrate, motility, indole, urea, and triple sugar iron tests), following which they were purified and aseptically inoculated into uniquely labeled 1 mL skim-milk tryptone glucose glycerol (STGG)-contained vials. Contaminants were defined as organisms not typically associated with bloodstream infections; these included non-pathogens and those more commonly associated with commensal skin bacteria. The STGG-contained isolates were stored temporarily at the sentinel laboratories and transferred (on ice) in batches to the Department of Medical Microbiology, UGMS (the reference laboratory) monthly.

Following the arrival of isolates at the reference laboratory, they were replated on blood agar (Oxoid, Basingstoke, Hants, UK), and their identities were confirmed using either the BD Bruker IVD MALDI-TOF version 4.2.100 (BRUKER Daltonics GmbH & Co. KG, Frahrenheitsteasse 4, 28359 Bremen, Germany) analyzer, the NMIC/ID 55 panel of BD Phoenix M50 ID/AST system (Becton Dickinson, Maryland, USA), or the bioMerieux VITEK 2 Compact 3D analyzer (Lab EQUIPMENT, bioMerieux, 100Rodolphe Street Durham, North Carolina 27716, USA). Subsequently, following standard procedures, antimicrobial susceptibility testing was done for the bacterial isolates using a combination of the BD Phoenix M50 analyzer and the Kirby-Bauer disk diffusion method and interpreted according to the Clinical and Laboratory Standards Institute (CLSI) [49] guidelines. *E. coli* ATCC 25922 was used as the control strain. The antibiotics tested were: ampicillin (4–16 µg/mL), ceftazidime (1–8 µg/mL), ceftriaxone (1–4 µg/mL), ciprofloxacin (0.0625–1 µg/mL), ertapenem (0.25–1 µg/mL), gentamicin (2–8 µg/mL), meropenem (0.125–8 µg/mL), sulfamethoxazole-trimethoprim (2/38–8/152 µg/mL), tetracycline (30 µg), azithromycin (15 µg), chloramphenicol (30 µg), nalidixic acid (30 µg), and cefotaxime (30 µg).

Suspensions of the isolates were prepared and adjusted to a turbidity identical to 0.5 McFarland standard (for the disk diffusion test) and 0.5–0.6 McFarland standard (for the BD Phoenix M50 analysis) using a nephelometer. In the disk diffusion tests, sterile cotton swabs were used to swab the suspensions onto Mueller Hinton agar (Oxoid, Basingstoke, Hants, UK) plates in a manner allowing for semi-confluent growth post-incubation. Antibiotic discs were placed on the seeded plates using antibiotic dispensers, and the plates were incubated at 37 °C for 18–24 h, after which the zones of inhibition around the antimicrobial discs were measured using a digital vernier caliper. Multidrug-resistant (MDR) phenotypes were defined relative to the panel of antibiotics reported as in vitro non-susceptibility to ≥1 agent in ≥3 antimicrobial classes [35]. Extended-spectrum beta-lactamase (ESBL) production was determined using the BD Phoenix M50 analyzer.

The bacterial isolates were stored at −80 °C at the Department of Medical Microbiology, University of Ghana Medical School, for future genomic analyses.

### 4.3. Data Analysis

Demographic, epidemiological, clinical, and microbiological information and AMR data in Gram-negative blood isolates were entered into a standardized Excel-based data entry sheet and imported into Statistical Package for the Social Sciences (SPSS), Version 25 for analysis. Descriptive statistics were used to compute the mean, standard deviation, median and interquartile range for continuous variables and frequencies and percentages for categorical variables. We calculated Cohen’s Kappa coefficient to assess the agreements between the sentinel and reference laboratories in the detection of the various Gram-negative blood isolates. AMR profiles were summarized for overall Gram-negative blood isolates and separately for NTS and *K. pneumoniae*, based on the detection in the reference laboratory. The percentage of isolates resistant, intermediate, or susceptible to each antimicrobial drug was calculated. Pooled prevalence rates of multidrug-resistant isolates were calculated. Differences in the prevalence of multidrug resistance per region, age group, gender, and healthcare facility were examined using the chi-square test. *p* < 0.05 was considered statistically significant.

### 4.4. Ethical Approval

Ethical clearance was obtained from the Ethical and Protocol Review Committee of the College of Health Sciences (Protocol Identification Number: CHS-Et/M.2-P 4.6/2021–2022).

## 5. Conclusions

In this pilot study, we demonstrated that in Ghana, as a model of low-income countries in sub-Saharan Africa, nationwide coordinated AMR surveillance in pathogens causing life-threatening BSIs in patients is practical and doable, depending on the provision of adequate support, expertise, and commitment. We also provided contemporary estimates on the extent to which NTS, *K. pneumoniae,* and other Gram-negative pathogens are involved in BSIs in Ghana. These AMR rates are rather alarming and require further verification in surveillance of longer duration to robustly estimate resistance rates to essential antibiotics. Such longer-duration surveillance studies or future routine surveillance programs are critical to determining seasonal or year-to-year variations in the occurrence of AMR. High resistance and multidrug resistance to first- or second-choice antibiotics, including penicillins, third-generation cephalosporins, and fluoroquinolones, were recorded, implying that these antibiotics might have limited effectiveness in BSI treatment. As a whole, our findings have major public health and clinical implications. We identified a major challenge at the sentinel sites involving the absence of key patient data. As this could potentially hinder effective AMR surveillance, healthcare facilities across the country need to ensure that patient data are completely filled out. Furthermore, there is an urgent need for the continuation of AMR surveillance with a longer duration in Gram-negative blood isolates and to expand such activities to other countries to provide reliable and robust evidence to guide clinical practice and policymaking.

## Figures and Tables

**Table 1:** Characteristics of the study participants.

		Number	%
Sentinel Site	37 Military Hospital	11	3.3
	Accra Regional Hospital	56	16.8
	Eastern Regional Hospital	41	12.3
	Effia Nkwanta Regional Hospital	22	6.6
	Komfo Anokye Teaching Hospital	34	10.2
	Korle Bu Teaching Hospital	93	27.8
	Princess Marie Louise Children’s Hospital	30	9
	Private Labs	31	9.3
	St. Patrick’s Hospital	9	2.7
	Volta Regional Hospital	7	2.1
City	Accra	221	66.2
	Effia Nkwanta	22	6.6
	Ho	7	2.1
	Koforidua	41	12.3
	Kumasi	34	10.2
	Maase-Offinso	9	2.7
Region	Ashanti	43	12.9
	Eastern	41	12.3
	Greater Accra	221	66.2
	Volta	7	2.1
	Western	22	6.6
Gender	Female	78	23.4
	Male	76	22.8
	Unknown	180	53.9
Age groups	under 5 years	85	25.0
	5–19 years	22	6.5
	20–59 years	55	16.2
	60+ years	18	5.3
	Unknown	160	47.1
Diagnosis	Bacteremia	33	9.9
	Sepsis	296	88.6
	Otitis media	1	0.3
	Severe anemia & malaria	1	0.3
	Urosepsis	2	0.6
	Not indicated	1	0.3

**Table 2:** Detection of Gram-negative bacteria at the sentinel and reference laboratories.

	Sentinel Laboratory		Reference Laboratory			
	Number	Percent	Number	Percent	Kappa Coefficient	*p* Value
*Acinetobacter baumannii*	23	6.9	40	12.0	0.669	<0.001
*Escherichia coli*	85	25.4	88	26.3	0.805	<0.001
*Klebsiella pneumoniae*	67	20.1	85	25.4	0.796	<0.001
*Pseudomonas aeruginosa*	22	6.6	25	7.5	0.840	<0.001
Non-typhoidal *Salmonella*	21	6.3	18	5.4	0.864	<0.001
Other	115	34.4	77	23.1		
Missing	1	0.3	1	0.3		
Total	334	100.0	334	100.0		

**Table 3:** Antimicrobial susceptibility of the isolates.

	All Gram-Negative Isolates	*Klebsiella pneumoniae*	Non-typhoidal *Salmonella*	*Acinetobacter baumannii*	*Escherichia coli*	*Pseudomonas aeruginosa* ^i^
Total	330	84	18	40	88	25
Meropenem	92 ^a^ (28.0%)	20 (23.8%)	3 (16.7%)	12 ^g^ (30.8%)	23 (26.1%)	13 (52.0%)
Tetracycline	251 (76.1%)	75 (89.3%)	6 (33.3%)	22 (55.0%)	71 (80.7%)	23 (92.0%)
Chloramphenicol	197 (59.7%)	59 (70.2%)	5 (27.8%)	35 (87.5%)	36 (40.9%)	19 (76.0%)
Gentamicin	157 ^b^ (53.8%)	63 ^e^ (82.9%)	4 ^f^ (23.5%)	14 ^g^ (45.2%)	34 ^e^ (44.7%)	6 (24.0%)
Cefotaxime	256 ^a^ (77.8%)	72 (85.7%)	7 (38.9%)	36 (90.0%)	64 ^h^ (73.6%)	21 (84.0%)
Azithromycin	138 ^c^ (42.1%)	39 (46.4%)	4 ^f^ (23.5%)	17 (42.5%)	41 (46.6%)	9 (36.0%)
Ciprofloxacin	177 ^a^ (55.3%)	62 (73.8%)	5 (27.8%)	15 ^g^ (44.1%)	54 (61.4%)	8 (32.0%)
Ceftriaxone	227 ^d^ (73.7%)	70 ^e^ (84.3%)	4 (22.2%)	24 ^g^ (88.9%)	67 ^h^ (77.9%)	20 (80.0%)
Ampicillin	293 ^a^ (89.3%)	83 ^e^ (100.0%)	6 (33.3%)	37 ^g^ (94.9%)	77 (87.5%)	24 (96.0%)
Ceftazidime	178 (56.3%)	58 (69.0%)	5 (27.8%)	19 ^g^ (57.6%)	59 (67.0%)	10 (40.0%)
Ertapenem	146 ^a^ (44.4%)	25 (29.8%)	3 (16.7%)	36 (90.0%)	26 (29.5%)	21 (84.0%)
Nalidixic acid	209 (63.3%)	49 (58.3%)	3 (16.7%)	20 (50.0%)	68 (77.3%)	23 (92.0%)
Trimethoprim-sulfamethoxazole	236 (71.5%)	72 (85.7%)	6 (33.3%)	23 (57.5%)	63 (71.6%)	23 (92.0%)
Multidrug resistant	290 (88.0%)	81 (96.4%)	7 (38.9%)	39 (97.5%)	83 (94.3%)	25 (100.0%)

^a^ Overall, 329 isolates were tested for meropenem, cefotaxime, ciprofloxacin, ampicillin, and ertapenem; ^b^ 296 isolates were tested against gentamicin; ^c^ 328 isolates were tested against azithromycin; ^d^ 323 isolates were tested against ceftriaxone; ^e^ 76 *K. pneumoniae* isolates were tested against gentamicin and 83 against ceftriaxone and ampicillin; ^f^ 17 NTS isolates were tested against gentamicin and azithromycin; ^g^ Of the *Acinetobacter baumannii* isolates, 39 were tested against meropenem, 31 against gentamicin and ampicillin, 34 against ciprofloxacin, 27 against ceftriaxone, and 33 against ceftazidime; ^h^ 87 of the *E. coli* isolates were tested against cefotaxime, and 86 against ceftriaxone; ^i^ Many of these antimicrobials wouldn’t be used clinically for *P. aeruginosa* because they are ineffective in vivo.

**Table 4:** Multidrug resistance among the Gram-negative isolates by background characteristics.

		Number Tested	Multidrug Resistant N (%)	*p*-Value
Sentinel Site	37 Military Hospital	11	9 (81.8)	0.315
	Accra Regional Hospital	56	45 (80.4)	
	Eastern Regional Hospital	41	36 (90.0)	
	Effia Nkwanta Regional Hospital	22	17 (81.0)	
	Komfo Anokye Teaching Hospital	34	33 (97.1)	
	Korle Bu Teaching Hospital	93	83 (91.2)	
	Princess Marie Louise Children’s Hospital	30	25 (83.3)	
	Private labs	31	26 (83.8)	
	St. Patrick’s Hospital	9	7 (100.0)	
	Volta Regional Hospital	7	7 (100.0)	
City	Accra	219	188 (85.8)	0.231
	Effia Nkwanta	21	17 (81.0)	
	Ho	7	7 (100.0)	
	Koforidua	40	36 (90.0)	
	Kumasi	34	33 (97.1)	
	Maase-Offinso	9	9 (100.0)	
Region	Ashanti	43	42 (97.7)	0.318
	Eastern	40	36 (90.0)	
	Greater Accra	219	188 (85.8)	
	Volta	7	7 (100.0)	
	Western	21	17 (81.0)	
Gender	Female	78	70 (89.7)	0.534
	Male	76	64 (86.5)	
Age	<5 years	85	74 (87.1)	0.962
	5–19 years	22	19 (86.4)	
	20–59 years	51	45 (88.2)	
	60+ years	18	15 (83.3)	

**Table 5:** Sentinel sites and corresponding laboratories.

Sentinel Sites	Reference Laboratory and Coordinating Centre
Greater Accra Regional Hospital	Department of Medical Microbiology, University of Ghana Medical School ^*^.
Princess Marie Louise Children’s Hospital	
Korle Bu Teaching Hospital *	
37 Military Hospital	
Eastern Regional Hospital, Koforidua ^*^	
Komfo Anokye Teaching Hospital	
Two private laboratories	
Bolgatanga Regional Hospital	
Volta Regional Hospital ^*^	

* These sites had been supported and upgraded by the Ghana Fleming Fund country grant.

## Data Availability

The data presented in this study are available upon reasonable request via dancohen@tauex.tau.ac.il or esampane-donkor@ug.edu.gh.

## Supplementary Materials

The following supporting information can be downloaded at: https://www.mdpi.com/article/10.3390/antibiotics12020255/s1, Table S1: Antimicrobial susceptibility of all Gram-negative blood isolates (N = 334); Table S2: Antimicrobial susceptibility of *K. pneumoniae* and non-typhoidal *Salmonella* isolates; Table S3: Antimicrobial susceptibility of *A. baumannii*, *E. coli* and *P. aeruginosa* isolates.
