# Supplementary material for: Multicenter Surveillance of Antimicrobial Resistance among Gram-Negative Bacteria Isolated from Bloodstream Infections in Ghana
Source: Antibiotics (Basel). 2023 Jan 27;12(2):255. doi: 10.3390/antibiotics12020255 (PMC9951917; doi:10.3390/antibiotics12020255)
Supplement: Supplementary file 1 [file antibiotics-12-00255-s001.zip › antibiotics-2168501-supplementary.pdf]

antibiotics

Article

Multicenter Surveillance of Antimicrobial Resistance among Gram-Negative Bacteria Isolated from Bloodstream Infections in Ghana

Eric S. Donkor [1,†], Khitam Muhsen [2,3,†], Sherry A. M. Johnson [4], Fleischer C. N. Kotey [1], Nicholas T. K. D. Dayie [1], Patience B. Tetteh-Quarcoo [1], Edem M. A. Tette [5], Mary-Magdalene Osei [1], Beverly Egyir [6], Nicholas I. Nii-Trebi [7], Godfred Owusu-Okyere [1,8], Alex Owusu-Ofori [9,10], Yonatan Amir [2], Saritte Perlman [2], Perdita Hilary Lopes [11], Adjo Mfodwo [11], Nicola C. Gordon [12], Louise Gresham [13], Mark Smolinski [13] and Dani Cohen [2,3,*]

1 Department of Medical Microbiology, University of Ghana Medical School, Accra 00233, Ghana
2 Department of Epidemiology and Preventive Medicine, School of Public Health, Sackler Faculty of Medicine, Tel Aviv University, Tel Aviv, 6139001, Israel
3 Middle East Consortium on Infectious Disease Surveillance (MECIDS), Jerusalem, 9149302, Israel.
4 School of Veterinary Medicine, University of Ghana, Accra 00233, Ghana
5 Department of Community Health, University of Ghana Medical School, Accra, 00233, P.O. Box KB 4236, Ghana
6 Department of Bacteriology, Noguchi Memorial Institute for Medical Research, University of Ghana, Accra 00233, Ghana
7 Department of Medical Laboratory Sciences, School of Biomedical & Allied Health Sciences, College of Health Sciences, University of Ghana, Accra 00233, Ghana
8 National Public Health Reference Laboratory, Accra 00233, Ghana
9 Department of Clinical Microbiology, Kwame Nkrumah University of Science and Technology, Kumasi 00233, Ghana
10 Directorate of Laboratory Services, Komfo Anokye Teaching Hospital, Kumasi, Ghana
11 Mott MacDonald, Accra, 00233, Ghana
12 Mott MacDonald, N11 3JB London, UK
13 Ending Pandemics, San Francisco, CA, USA
* Correspondence: dancohen@tauex.tau.ac.il; Tel.: +972-3-6407081
† These authors contributed equally to this work.

Supplementary Tables

Supplementary Table S1. Antimicrobial susceptibility of all Gram-negative blood isolates (N=334)

	**Resistant N (%)**	**Intermediate N (%)**	**Susceptible N (%)**
**Meropenem**	92 (28.0%)	12 (3.6%)	225 (68.4%)
**Tetracycline**	251 (76.1%)	4 (1.2%)	75 (22.7%)
**Chloramphenicol**	197 (59.7%)	13 (3.9%)	120 (36.4%)
**Gentamicin**	157 (53.8%)	7 (2.4%)	128 (43.8%)
**Cefotaxime**	256 (77.8%)	17 (5.2%)	56 (17.0%)
**Azithromycin**	138 (42.1%)	4 (1.2%)	186 (56.7%)
**Ciprofloxacin**	177 (55.3%)	30 (9.4%)	113 (35.3%)
**Ceftriaxone**	227 (73.7%)	7 (2.3%)	74 (24.0%)
**Ampicillin**	293 (89.3%)	7 (2.1%)	28 (8.5%)
**Ceftazidime**	178 (56.3%)	30 (9.5%)	108 (34.2%)
**Ertapenem**	146 (44.4%)	14 (4.3%)	169 (51.4%)
**Nalidixic acid**	209 (63.3%)	29 (8.8%)	92 (27.9%)
**Trimethoprim-sulfamethoxazole**	236 (71.5%)	1 (0.3%)	93 (28.2%)
**Multidrug resistant**	290 (88.0%)		
**Susceptible to all antibiotics tested**			5 (1.5%)

Supplementary Table S2. Antimicrobial susceptibility of *K. pneumoniae* and non-typhoidal *Salmonella* isolates

	No. tested isolates	Resistant N (%)	Intermediate N (%)	Susceptible N (%)
Antimicrobial susceptibility of *Klebsiella pneumoniae* blood isolates				
**Meropenem**	84	20 (23.8%)	4 (4.8%)	60 (71.4%)
**Tetracycline**	84	75 (89.3%)	0 (0.0%)	9 (10.7%)
**Chloramphenicol**	84	59 (70.2%)	1 (1.2%)	24 (28.6%)
**Gentamicin**	76	63 (82.9%)	1 (1.3%)	12 (15.8%)
**Cefotaxime**	84	72 (85.7%)	2 (2.4%)	10 (11.9%)
**Azithromycin**	84	39 (46.4%)	2 (2.4%)	43 (51.2%)
**Ciprofloxacin**	84	62 (73.8%)	8 (9.5%)	14 (16.7%)
**Ceftriaxone**	83	70 (84.3%)	1 (1.2%)	12 (14.5%)
**Ampicillin**	83	83 (100.0%)	0 (0.0%)	0 (0.0%)
**Ceftazidime**	84	58 (69.0%)	9 (10.7%)	17 (20.2%)
**Ertapenem**	84	25 (29.8%)	6 (7.1%)	53 (63.1%)
**Nalidixic acid**	84	49 (58.3%)	13 (15.5%)	22 (26.2%)
**Trimethoprim-sulfamethoxazole**	84	72 (85.7%)	1 (1.2%)	11 (13.1%)
**Multidrug resistant**	84	81 (96.4%)		
**Susceptible to all antibiotics tested**	84			0 (0.0%)
Antimicrobial susceptibility of Non-typhoidal *Salmonella* blood isolates				
**Meropenem**	18	3 (16.7%)	1 (5.6%)	14 (77.8%)
**Tetracycline**	18	6 (33.3%)	0 (0.0%)	12 (66.7%)
**Chloramphenicol**	18	5 (27.8%)	0 (0.0%)	13 (72.2%)
**Gentamicin**	17	4 (23.5%)	0 (0.0%)	13 (76.5%)
**Cefotaxime**	18	7 (38.9%)	4 (22.2%)	7 (38.9%)
**Azithromycin**	17	4 (23.5%)	0 (0.0%)	13 (76.5%)
**Ciprofloxacin**	18	5 (27.8%)	0 (0.0%)	13 (72.2%)
**Ceftriaxone**	18	4 (22.2%)	0 (0.0%)	14 (77.8%)
**Ampicillin**	18	6 (33.3%)	0 (0%)	12 (66.7%)
**Ceftazidime**	18	5 (27.8%)	1 (5.6%)	12 (66.7%)
**Ertapenem**	18	3 (16.7%)	0 (0.0%)	15 (83.3%)
**Nalidixic acid**	18	3 (16.7%)	0 (0.0%)	15 (83.3%)
**Trimethoprim-sulfamethoxazole**	18	6 (33.3%)	0 (0.0%)	12 (66.7%)
**Multidrug resistant**	18	7 (38.9%)		
**Susceptible to all antibiotics tested**	18			3 (16.7%)

Supplementary Table S3: Antimicrobial susceptibility of *A. baumannii, E. coli* and *P. aeruginosa* isolates

	**No. tested isolates**	**Resistant N (%)**	**Intermediate N (%)**	**Susceptible N (%)**
Antimicrobial susceptibility of *Acinetobacter baumannii* blood isolates				
**Meropenem**	39	12 (30.8%)	2 (5.1%)	25 (64.1%)
**Tetracycline**	40	22 (55.0%)	2 (5.0%)	16 (40.0%)
**Chloramphenicol**	40	35 (87.5%)	1 (2.5%)	4 (10.0%)
**Gentamicin**	31	14 (45.2%)	1 (3.2%)	16 (51.6%)
**Cefotaxime**	40	36 (90.0%)	1 (2.5%)	3 (7.5%)
**Azithromycin**	40	17 (42.5%)	-	23 (57.5%)
**Ciprofloxacin**	34	15 (44.1%)	7 (20.6%)	15 (44.1%)
**Ceftriaxone**	27	24 (88.9%)	1 (3.7%)	2 (7.4%)
**Ampicillin**	39	37 (94.9%)	1 (2.6%)	1 (2.6%)
**Ceftazidime**	33	19 (57.6%)	4 (12.1%)	10 (30.3%)
**Ertapenem**	40	36 (90.0%)	1 (2.5%)	3 (7.5%)
**Nalidixic acid**	40	20 (50.0%)	7 (17.5%)	13 (32.5%)
**Trimethoprim-sulfamethoxazole**	40	23 (57.5%)	-	17 (42.5%)
**Multidrug resistant**	40	39 (97.5%)		
**Susceptible to all antibiotics tested**	40			0 (0.0%)
Antimicrobial susceptibility of *E. coli* blood isolates				
**Meropenem**	88	23 (26.1%)	-	65 (73.9%)
**Tetracycline**	88	71 (80.7%)	-	17 (19.3%)
**Chloramphenicol**	88	36 (40.9%)	5 (5.7%)	47 (53.4%)
**Gentamicin**	76	34 (44.7%)	2 (2.6%)	40 (52.6)
**Cefotaxime**	87	64 (73.6%)	6 (6.9%)	17 (19.5%)
**Azithromycin**	88	41 (46.6%)	1 (1.1%)	46 (52.3%)
**Ciprofloxacin**	88	54 (61.4%)	9 (10.2%)	25 (28.4%)
**Ceftriaxone**	86	67 (77.9%)	2 (2.3%)	17 (19.8%)
**Ampicillin**	88	77 (87.5%)	3 (3.4%)	8 (9.1%)
**Ceftazidime**	88	59 (67.0%)	8 (9.1%)	21 (23.9%)
**Ertapenem**	88	26 (29.5%)	3 (3.4%)	59 (67.0)
**Nalidixic acid**	88	68 (77.3%)	3 (3.4%)	17 (19.3%)
**Trimethoprim-sulfamethoxazole**	88	63 (71.6%)	-	25 (28.4%)
**Multidrug resistant**	88	83 (94.3%)		
**Susceptible to all antibiotics tested**	88			2 (2.3%)
Antimicrobial susceptibility of *Pseudomonas aeruginosa* blood isolates				
**Meropenem**	25	13 (52.0%)	-	12 (48.0%)
**Tetracycline**	25	23 (92.0%)	1 (4.0%)	1 (4.0%)
**Chloramphenicol**	25	19 (76.0%)	2 (8.0%)	4 (16.0%)
**Gentamicin**	25	6 (24.0%)	-	19 (76.0%)
**Cefotaxime**	25	21 (84.0%)	-	4 (16.0%)
**Azithromycin**	25	9 (36.0%)	-	16 (64.0%)
**Ciprofloxacin**	25	8 (32.0%)	2 (8.0%)	15 (60.0%)
**Ceftriaxone**	25	20 (80.0%)	3 (12.0%)	2 (8.0%)
**Ampicillin**	25	24 (96.0%)	-	1 (4.0%)
**Ceftazidime**	25	10 (40.0%)	-	15 (60.0%)
**Ertapenem**	25	21 (84.0%)	1 (4.0%)	3 (12.0%)
**Nalidixic acid**	25	23 (92.0%)	-	2 (8.0%)
**Trimethoprim-sulfamethoxazole**	25	23 (92.0%)	-	2 (8.0%)
**Multidrug resistant**	25	25 (100%)		
**Susceptible to all antibiotics tested**	25			0 (0%)
